# Supplementary material for: A Fluorescent Reporter Virus Toolkit for Interrogating Enterovirus Biology and Host Interactions
Source: Viruses. 2025 May 30;17(6):796. doi: 10.3390/v17060796 (PMC12197625; doi:10.3390/v17060796)
Supplement: Supplementary file 1 [file viruses-17-00796-s001.zip › Figure S1.pdf]

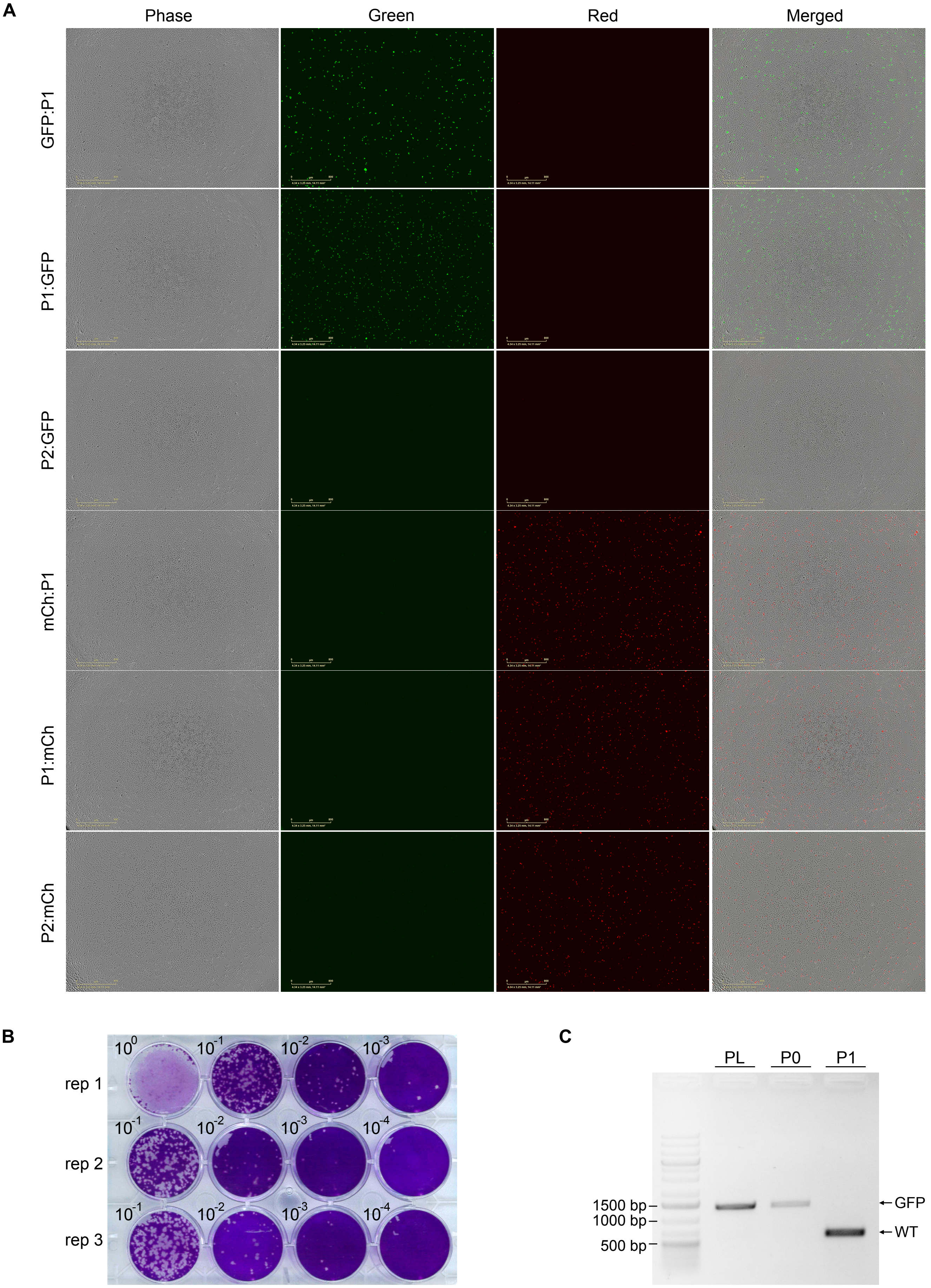

Supplementary Figure S1. Titration and validation of CVB3 reporter viruses. (A) Fluorescence and brightfield images of HeLa-H1 cells infected with each of the CVB3 reporter viruses, taken at 8 hpi. **(B)** Plaque assay of passage 0 CVB3 P2:GFP in HeLa-H1 cells. The corresponding dilutions are indicated above each well for each of the three replicates (rep1-3). (B) RT-PCR from the plasmid (PL), passage 0 (P0), or passage 1 (P1) of the P2:GFP reporter, amplifying a region flanking the eGFP insertion site. The expected amplicon size is 1373 bp when eGFP is present, and 605 bp when absent (indicated by arrows).
